# The Feasibility and Histological Diagnostic Accuracy of Novel Menghini Needle (EUS Sonopsy CY™) for Endoscopic Ultrasound-Guided Fine-Needle Aspiration Biopsy of Solid Pancreatic Masses: A Prospective Crossover Study Comparing Standard Biopsy Needles

**DOI:** 10.1155/2019/5810653

**Published:** 2019-10-21

**Authors:** Ryo Igarashi, Atsushi Irisawa, Manoop S. Bhutani, Irina M. Cazacu, Goro Shibukawa, Ai Sato, Akane Yamabe, Takumi Maki, Yoshitsugu Yoshida, Shogo Yamamoto, Tsunehiko Ikeda, Hiroshi Hojo

**Affiliations:** ^1^Department of Gastroenterology, Aizu Medical Center, Fukushima Medical University, Fukushima, Japan; ^2^Department of Gastroenterology, Dokkyo Medical University, Tochigi, Japan; ^3^Department of Gastroenterology, Hepatology and Nutrition, Unit 1466, MD Anderson Cancer Center, University of Texas, Houston, Texas, USA; ^4^Department of Pathology, Aizu Medical Center, Fukushima Medical University, Fukushima, Japan

## Abstract

**Background and Objectives:**

Recently, a 21G Menghini-type needle for EUS-guided fine-needle aspiration biopsy (EUS-FNAB) has been developed. The stylet of the EUS Sonopsy CY™ remains inside the needle during aspiration. Therefore, it is expected to obtain higher-quality histological core specimens without crushing the material or blood contamination. The aim of this study is to evaluate the feasibility and diagnostic accuracy of EUS-FNAB of solid pancreatic masses with this new biopsy needle.

**Methods:**

A total of 30 patients with solid pancreatic masses who underwent EUS-FNAB with two different types of needles, EUS Sonopsy™ and ProCore™, were included in a prospective, randomized, controlled, crossover study. All the pancreatic masses were punctured with the two needles and were randomized regarding the order of the needle to be used. The primary outcome was to compare the diagnostic accuracy and the rates of tissue acquisition of the two needles.

**Results:**

The tissue acquisition rate was not significantly different between the EUS Sonopsy CY™ needle and the ProCore™ needle (78.6% vs. 82.1%, *P* = 1.00). The histological diagnostic accuracy was also similar between the two needles (73% vs. 80%, *P* = .63). There was also no difference regarding the accuracy of cytology alone and the combination of both histological and cytological assessments between the EUS Sonopsy CY™ needle and the ProCore™ needle (90% vs. 87%, *P* = 1.00 and 90% vs. 90%, *P* = 1.00, respectively).

**Conclusions:**

EUS Sonopsy CY™ is a reliable device for EUS-FNAB of solid pancreatic masses.

## 1. Introduction

An endoscopic ultrasound-guided fine-needle aspiration biopsy (EUS-FNAB) is a validated and recommended technique for tissue diagnosis of pancreatic masses [1]. Previous studies have shown that EUS-FNA is very accurate for the diagnosis of pancreatic lesions, with a sensitivity of 78–95%, specificity of 75–100%, and a total diagnostic accuracy of 78–95% [2–4].

Tissue histology may be required to establish a definitive diagnosis, especially when FNA cytological assessment alone is inconclusive. However, sometimes, it is difficult to obtain enough tissue for histological examination using conventional EUS-FNA needle [5]. Core specimens with preserved architecture are crucial to diagnose and fully characterize certain neoplasms, such as lymphomas, neuroendocrine neoplasms, and gastrointestinal stromal tumors. Moreover, unlike cytological aspirates, examination of core specimens facilitates the diagnosis of benign diseases such as autoimmune pancreatitis [6]. Furthermore, using core-biopsy samples, tissue profiling to guide targeted therapies for individualized treatment of patients with certain gastrointestinal cancers can be performed [7, 8].

**Figure 1:**
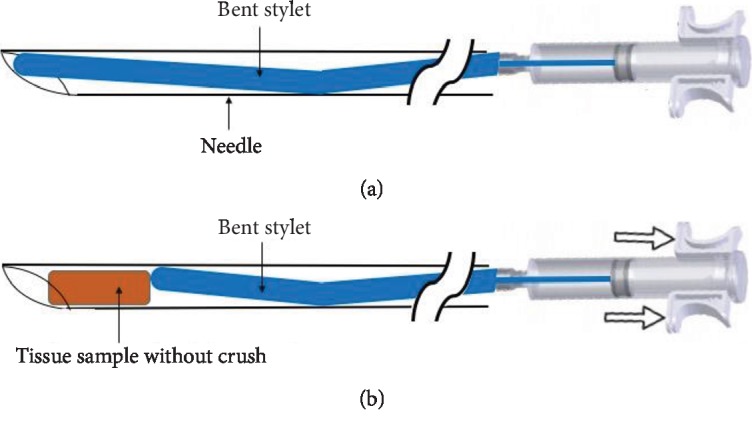
The structure and features of EUS Sonopsy CY™. (a) The suction piston is connected to a bent stylet. (b) When the aspiration is performed using the suction piston, the stylet remains inside the needle during aspiration which create the tiny space for trapping of the aspirated material.

In order to obtain a larger amount of material from the targeted lesions, a biopsy needle with reverse-bevel technology (EchoTip ProCore™; Cook Medical, Bloomington, USA) was developed. However, a recent meta-analysis has shown that there is no significant difference between the ProCore™ and standard FNA needles regarding sample adequacy, diagnostic accuracy, or tissue acquisition, whereas the ProCore™ required fewer needle passes [9]. Recently, various needles such as SharkCore™ (Medtronic, Minneapolis, USA) and the Acquire™ needle (Boston Scientific, Marlborough, USA) have been developed to obtain a greater amount of tissue and achieve a more accurate diagnostic rate [10, 11]. EUS Sonopsy CY™ (Hakko Co., Tokyo, Japan) is a newly designed biopsy aspiration needle. This needle features the Menghini-type needle tip biopsy system, a needle shape suitable for biopsy, and good supersonic wave depiction characteristics. In particular, the mechanism of EUS Sonopsy CY™ is very unique as the stylet remains inside the needle during aspiration (Figures 1(a) and 1(b)). Therefore, it is expected to obtain adequate tissue samples without crushing the material or contaminating with blood. The aim of this study is to evaluate the feasibility and diagnostic yields of this new biopsy needle in performing EUS-FNAB of solid pancreatic masses.

## 2. Materials and Methods

### 2.1. Study Design

A single-center, prospective, randomized controlled trial was conducted. It was designed as a crossover investigation between EUS Sonopsy CY™ and the ProCore™ needle for each pancreatic lesion. The study was approved by the Institutional Review Board of Fukushima Medical University, and written informed consent was obtained for this study.

The results of EUS-FNAB by using EUS Sonopsy CY™ and the ProCore™ needle were compared. The primary outcome was to compare the histological diagnostic accuracy and the rates of tissue acquisition of the two needles. The secondary outcomes were to compare the accuracy of cytological diagnosis, the accuracy of EUS-FNAB diagnosis (the combination of histology and cytology), and adverse event rates for the two needles. The final diagnosis was based on surgical resection or clinical follow-up for at least 6 months after EUS-FNA. In addition, we have analyzed the total cost of all used needles in each patient to compare between 2 groups. The cost was converted into US dollars using the most recent annual exchange rate published by the OECD (1 USD = 110.42 JPY), accessed 17 Aug 2018.

### 2.2. Patient/Study Population

Consecutive patients undergoing EUS-FNAB of a solid pancreatic mass were prospectively enrolled in this study from May 2015 to March 2017. The inclusion criteria were (1) 20 years of age and older, (2) the ability to provide informed consent, and (3) the presence of a solid pancreatic mass detected by a CT scan or magnetic resonance imaging that could be safely punctured from the stomach or duodenum. The exclusion criteria were (1) an Eastern Cooperative Oncology Group performance status of 4, (2) American Society of Anesthesiologists Physical Status classification greater than 3, (3) continuous use of antithrombogenic agents, (4) pregnancy, (5) the inability to undergo an endoscopic exam, (6) diagnosis already known through other investigations, (7) presence of a coagulopathy (prothrombin time/international normalized ratio > 1.5), and (8) presence of thrombocytopenia (platelet count < 50, 000/mL).

### 2.3. Procedure/Intervention

EUS-FNAB was performed using a curved linear array echoendoscope (GF-UCT240 or GF-UCT260; Olympus Medical Systems, Tokyo, Japan). All procedures were performed at the participating facility by an experienced endosonographer (>100 EUS-FNA procedures). EUS-FNAB was performed in the left lateral decubitus position under moderate conscious sedation using intravenous injection of midazolam and pentazocine. For each lesion, four passes were performed, two with the EUS Sonopsy CY™ (S) needle and two with the ProCore™ 22 G needle (P). The patients were randomly assigned in the order of S-P-S-P or P-S-P-S. For pathological examination, the material obtained by EUS-FNAB was expressed on glass slides or placed in a container by pushing air from a syringe. Most of the tissue specimens were immediately fixed in 10% neutral-buffered formalin solution for histological examination. The other specimens were used for cytopathological examination.

### 2.4. Pathological Assessment

All samples were evaluated by a single experienced pathologist who was blinded to the information regarding needle selection. The histological diagnosis was determined based on hematoxylin and eosin staining and additional immunohistochemistry if needed.

### 2.5. Statistical Analysis

It was reported that the accuracy of histological diagnosis by using a 22-gauge needle in 2 FNA procedures for pancreatic masses was 62.5%. Based on previous studies, the accuracy of histological diagnosis was estimated at 65% for the ProCore™ group. However, there is no relevant data regarding this aspect of EUS Sonopsy CY™. We estimated the accuracy of histological diagnosis at 85% for EUS Sonopsy CY™ based on our clinical experience. A 2-tailed sample-size calculation was performed with the type I error rate (*α*) set at .05 to attain 80% power to detect a difference of 20% in the accuracy of histological diagnosis. It resulted in target sample sizes of 82 patients for each method. Continuous variables pertaining to baseline characteristics were presented as the mean SD and range. The diagnostic accuracy was compared by using the McNemar test. All analyses were conducted using Stata 14.0™ for Windows (Stata Corp., TX, USA). A *P* value less than .05 was considered to indicate a statistically significant difference.

## 3. Results

**Figure 2:**
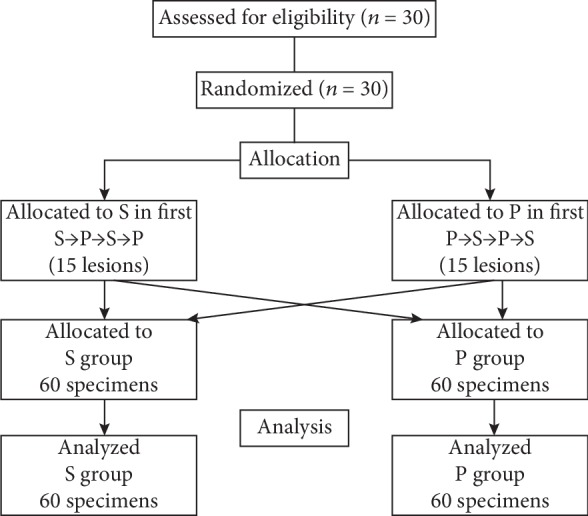
Flow diagram of the trial.

Between May 2015 and March 2017, 30 patients with solid pancreatic masses (11 men and 19 women; mean age, 74.4 years) were enrolled in this study (Figure 2). The characteristics of patients and the final diagnosis are shown in Table 1.

In two patients, the EUS-FNAB procedure with the EUS Sonopsy CY™ needle using a transduodenal approach was not successful because it was impossible to take out the needle from the tip of the echoendoscope in the duodenal lumen.

Rates of histological core specimen acquisition were not significantly different between the EUS Sonopsy CY™ and the ProCore™ needles (78.6% vs. 82.1%, *P* = 1.00). The overall diagnostic accuracy of histological examination with a total of 4 passes (2 passes by EUS Sonopsy CY™ and 2 passes by the ProCore™) was 83% (25/30). In 5 patients, it was necessary to perform an additional puncture, because the tissue samples obtained within 4 passes had insufficient material for histopathological diagnosis. Regarding the separate diagnostic accuracy of histological examination, there was also no significant difference between EUS Sonopsy CY™ and the ProCore™ needles (73% vs. 80%, *P* = .63). The diagnostic accuracy of cytology alone and of the combination between both histological and cytological assessments were also similar between the two needles (90% vs. 87%, *P* = 1.00 and 90% vs. 90%, *P* = 1.00, respectively) (Table 2). In the subgroup analysis regarding the location of the echoendoscope, transgastric approach (*n* = 19, 63%) or transduodenal approach (*n* = 11, 37%), the rate of diagnostic accuracy for histology was also similar between the EUS Sonopsy CY™ and the ProCore™ (68% vs. 74%, *P* = 1.00 and 82% vs. 91%, *P* = 1.00, respectively). There were no procedure-related adverse events.

In addition, we have done the comparative analysis regarding the total cost of all used needles in each patient between 2 groups. The EUS Sonopsy CY™ group had a lower cost per patient than the ProCore™ group (average costs in each patient were 21,000 JPY (190 USD) vs. 30,000 JPY (272 USD), respectively).

## 4. Discussion

Although many EUS-FNAB needles have been developed, there is no uniform consensus regarding needle selection. This is the first report conveying the clinical value of EUS Sonopsy CY™ needle in patients with solid pancreatic masses. According to our results, there was no significant difference between the EUS Sonopsy CY™ and the ProCore™ needle. Consequently, this new needle proved to be noninferior compared to the previous EUS-FNAB needles. We can justify its use in terms of costs, even if we cannot puncture 2 cases in 30 cases because of the stiffness of the needle.

Various techniques and equipment have been studied to improve the diagnostic yield of EUS-FNAB. Multiple factors may contribute to the results of this procedure, such as the selection of needle, the sampling technique, and specimen handling/processing [12, 13]. Generally, it is important to increase the specimen cellularity and to decrease the blood contamination for achievement of higher diagnostic accuracy. Bhutani et al. showed that intermittent suction with smaller syringes (5-10 ml) provides optimal cellularity in EUS-guided FNA of mediastinal lymph nodes [14]. The European Society of Gastrointestinal Endoscopy technical guidelines recommended using suction for EUS-FNA of solid masses/cystic lesions but not for EUS-FNA of lymph nodes [15]. However, the suction technique may also increase the risk of structure damage and blood contamination which makes pathological evaluation difficult [16, 17]. Recently, the slow-pull technique has been introduced as a new sampling technique in EUS-FNA for pancreatic solid mass [18–20]. The slow-pull technique provides minimum negative pressure by removing the stylet from the needle slowly and continuously [21]. Moreover, the wet suction technique was also performed to enhance tissue acquisition by applying principles of fluid dynamics to the aspiration technique. The wet suction technique can potentially provide higher negative pressure to the needle tip when suction is applied to the syringe [22]. In a randomized controlled trial comparing the wet technique to standard suction FNA in patients with various solid masses and lymph nodes, the wet suction technique improved sample adequacy and quality [23]. Based on the hypothesis of the slow-pull technique or the wet suction technique, it was considered that EUS Sonopsy CY™ could also reduce the risk of structure damage and blood contamination, as the stylet remains inside the needle during aspiration.

In two cases, the pancreatic masses could not be punctured using EUS Sonopsy CY™ via a transduodenal approach because of the stiffness of the needle. It is made of stainless steel, being more rigid in comparison with other needles, and therefore, it was extremely difficult to puncture the pancreatic lesion with the scope in angulated position at the duodenum. However, when the procedure was technically feasible, histological tissue specimens were obtained in all cases. Accordingly, EUS Sonopsy CY™ may be considered as a first-line device for EUS-FNAB performed through the gastric/esophageal wall without strong angulation.

In this study, there was no difference between the two needles, although we expected to obtain better quality samples using the EUS Sonopsy CY™ in comparison with the ProCore™ needle due to the unique system of the EUS Sonopsy CY™. This may be caused by the small number of patients included in the study and also by the high sensitivity/specificity of both devices. In terms of diagnosis of pancreatic cancer, a small tissue specimen is enough.

The main advantage of histological core biopsies is that the specimens preserve their morphological architecture which makes the interpretation more accurate compared to cytological assessment. This is important for the diagnosis of well-differentiated adenocarcinomas because of the common cytological features between neoplastic and reactive ductal epitheliums. Moreover, a definitive diagnosis is frequently challenging for pathologists because cytological aspirates are often tiny, scant, and bloody [24–26]. This may result in unnecessary resection for benign disease or can cause delay in treatment for pancreatic cancer patients. Furthermore, histological core specimens are important for EUS-guided tissue acquisition because in most centers, a cytopathologist is not present on site. Consequently, the diagnostic accuracy can be improved by obtaining high-quality core specimens.

Current advances in basic medical sciences have enabled the development and clinical application of molecular targeting agents for various types of cancers [27]. The rapidly expanding knowledge of the molecular alterations of pancreatic cancer is providing new targets for disease characterization and early diagnosis [28]. There is a great expectation that molecular tests could substantially improve the early diagnosis as well as open new therapeutic possibilities for this aggressive disease [29]. Advances in molecular diagnostic techniques have made it possible to carry out various types of immunostaining and gene analyses using the material obtained by EUS-FNAB. Accordingly, accurately obtaining large tissue specimens by EUS-FNAB will be more important in the future.

Several limitations of this study should be noted. First, the target number of patients was not reached, because we could not enroll enough cases for a limited period of time in only one institution. However, in that situation, we could show no difference regarding the accuracy of cytology alone and the combination of both histological and cytological assessments between the EUS Sonopsy CY™ needle and the ProCore™ needle. Second, this study was single-blind only to the pathologists. However, we focused on the histological assessment and evaluated slides from both EUS Sonopsy CY™ and the ProCore™ groups for each lesion by using the crossover method to eliminate any selection bias of the lesions. Third, there are no uniform criteria for assessing the quality and quantity of the histological specimen obtained by EUS-FNAB.

## 5. Conclusion

Our study showed that EUS Sonopsy CY™ is a reliable device for EUS-guided FNAB of pancreatic solid mass. Current results do not demonstrate a significant difference between the EUS Sonopsy™ and the ProCore™ needles regarding diagnostic accuracy or histological core specimen acquisition. However, the advantage of EUS Sonopsy CY™ was shown in terms of cost. Larger, prospective randomized comparative studies are needed to confirm these findings.

## Figures and Tables

**Table 1:** Patient characteristics and final diagnoses.

	Pancreatic masses (*N* = 30)
Age (y), mean (SD)	74.4 (9.0)
Range	62-95
Sex	
Male	11
Female	19
Site of pancreatic mass	
Head	13
Body	12
Tail	5
Puncture route	
Transgastric	19
Transduodenal	11
Size of masses on EUS (mm), mean (SD)	27.6 (10.7)
Range	12-55
Final diagnosis	
Pancreatic adenocarcinoma	25
IPMC	1
Secondary tumors of the pancreas	1
Sarcoidosis	1
Autoimmune pancreatitis	1
Mass-forming pancreatitis	1

**Table 2:** Diagnostic accuracy of EUS-FNAB by using EUS Sonopsy CY™ (S group) and the ProCore™ needle (P group).

	S group	P group	*P* value
Histological examination			

All cases (*N* = 30)	73% (22/30)	80% (24/30)	0.63
Transgastric (*N* = 19)	68% (13/19)	74% (14/19)	1.00
Transduodenal (*N* = 11)	82% (9/11)	91% (10/11)	1.00

Cytological examination			

All cases (*N* = 30)	90% (27/30)	87% (26/30)	1.00
Histological and cytological examination			

All cases (*N* = 30)	90% (27/30)	90% (27/30)	1.00

## Data Availability

The data used to support the findings of this study are included within the article.
